# *Acinetobacter baumannii*: assessing susceptibility patterns, management practices, and mortality predictors in a tertiary teaching hospital in Lebanon

**DOI:** 10.1186/s13756-023-01343-8

**Published:** 2023-11-29

**Authors:** Rania Itani, Hani M. J. Khojah, Samar Karout, Deema Rahme, Lara Hammoud, Reem Awad, Rana Abu-Farha, Tareq L. Mukattash, Hamza Raychouni, Abdalla El-Lakany

**Affiliations:** 1 Pharmacy Practice Department, Faculty of Pharmacy, Beirut Arab University, Riad El Solh, 1107 2809, P.O. Box: 11-5020, Beirut, Lebanon; 2 Department of Pharmacy Practice, College of Pharmacy, Taibah University, P.O. Box: 30051, 41477 Madinah, Kingdom of Saudi Arabia; 3 INSPECT-LB (Institut National de Santé Publique, d’Épidémiologie Clinique et de Toxicologie-Liban), Beirut, Lebanon; 4 Pharmacy Department, Hammoud Hospital University Medical Center, Sidon, Lebanon; 5 Department of Clinical Pharmacy and Therapeutics, Faculty of Pharmacy, Applied Science Private University, P.O. Box: 11931, Amman, Jordan; 6 Department of Clinical Pharmacy, Faculty of Pharmacy, Jordan University of Science and Technology, P.O. Box: 3030, Irbid, 22110 Jordan; 7 Intensive Care Unit, Central Military Hospital, Military Healthcare, Lebanese Army, Beirut, Lebanon; 8 Intensive Care Unit, American University of Beirut Medical Center, Beirut, Lebanon; 9 Department of Pharmaceutical Sciences, Faculty of Pharmacy, Beirut Arab University, Riad El Solh, 1107 2809, P.O. Box: 11-5020, Beirut, Lebanon; 10 Department of Pharmacognosy, Faculty of Pharmacy, Alexandria University, Alexandria, Egypt

**Keywords:** *Acinetobacter baumannii*, Susceptibility patterns, Multidrug resistance, Antibiotics, Risk factors, Treatment outcomes, Mortality, Lebanon

## Abstract

**Background:**

*Acinetobacter baumannii* is a major nosocomial pathogen capable of causing life-threatening infections. This bacterium is highly resistant to antibiotics and associated with high mortality rates. Therefore, this study aimed to evaluate *A. baumannii*'s susceptibility patterns to antimicrobials, assess the appropriateness of the initiated antimicrobial therapy, determine the mortality rate, and identify predictors associated with mortality.

**Methods:**

A retrospective observational study was conducted among patients infected with *A. baumannii* at a university hospital in Lebanon through the revision of medical records. Kaplan–Meier survival analysis and log-rank tests were used to analyze time-to-mortality. Binary logistic regression was performed to identify predictors of mortality.

**Results:**

The records of 188 patients were screened, and 111 patients with *A. baumannii* infection were enrolled. Almost all isolates were resistant to carbapenem, and 43% of the isolates were extensively-drug resistant. Almost half of the patients received initial inappropriate antimicrobial therapy (n = 50, 45.1%). The 30-day mortality rate associated with *A. baumannii* infection was 71.2% (79/111). The time to mortality in patients who received inappropriate antimicrobial therapy (5.70 ± 1.07 days) was significantly shorter than in those who received appropriate antimicrobial therapy (12.43 ± 1.01 days, *P* < 0.01). Binary logistic regression revealed that inappropriate antimicrobial therapy (adjusted odds ratio [AOR] = 16.22, 95% CI 2.68–9.97, *P* = 0.002), mechanical ventilation (AOR = 14.72, 95% CI 3.27–6.61, *P* < 0.001), and thrombocytopenia (AOR = 8.82, 95% CI 1.12–9.75, *P* = 0.003) were more likely associated with mortality.

**Conclusions:**

*A. baumannii* exhibits an alarming mortality rate among infected patients. Thrombocytopenia, mechanical ventilation, and inappropriate antibiotic administration are associated with mortality in patients infected with *A. baumannii*. The prompt initiation of appropriate antimicrobial therapy, infection control measures, and effective stewardship program are crucial to reduce the incidence of *A. baumannii* and improve the treatment outcomes.

## Background

*Acinetobacter baumannii* is a gram-negative coccobacillus that poses a significant public health threat, particularly among critically ill patients, and leads to life-threatening infections with poor prognosis and high mortality rate [1, 2]. Moreover, this bacterium has developed a remarkable resistance to a wide range of antibiotics [3, 4].

*Acinetobacter baumannii* can cause a variety of infections, mostly involving the respiratory tract, especially ventilator-associated pneumonia and catheter-associated bacteremia [3]. *Acinetobacter baumannii* is an opportunistic pathogen that usually colonizes and survives in the respiratory airways, as it has a strong ability to adhere to epithelial cells in the respiratory tract and form biofilms, which makes it resistant to antimicrobials [4]. It is considered among the most common pathogens detected in sputum cultures and endotracheal aspirates among long-term hospitalized patients [5]. Furthermore, *A. baumannii* colonizes mechanical equipment including respiratory support equipment, suction devices, and feeding tubes, and it can easily spread through the vicinity of infected patients [6]. For this reason, the World Health Organization (WHO) issued an updated guidance for cleaning and disinfecting respiratory equipment [7].

*Acinetobacter baumannii* isolates have been frequently found to possess multidrug resistance (MDR), extensive drug resistance (XDR), or even pandrug resistance (PDR) [8, 9]. This pathogen commonly causes nosocomial infections that are common among critically ill patients admitted to the intensive care unit and among immunocompromised patients [8, 10]. Infections associated with the acquaintance of this pathogen include urinary tract infections, wound infections, pneumonia, and bacteremia [11]. These infections result in a longer hospital stay and a higher risk of hospital death [12].

Nominated as a pathogen of "high priority" by the World Health Organization (WHO), *A. baumannii* exhibits unique patterns regarding pathogenesis, transmissibility, and mechanisms of resistance. Its exceptional ability to acquire resistance against commonly used hospital antibiotics, coupled with its high survival rate and rapid spread within the hospital environment, makes it a formidable microorganism [11, 13]. *A. baumannii* belongs to the group of ESKAPE pathogens (*Enterococcus faecium, Staphylococcus aureus, Klebsiella pneumoniae, A. baumannii, Pseudomonas aeruginosa,* and *Enterobacter* species), which includes microorganisms responsible for a significant proportion of nosocomial infections worldwide and possess the ability to evade the effects of antimicrobials [14, 15].

In the last three decades, *A. baumannii* has contributed to the high global morbidity and mortality rate, ranging between 26.5% and 91%, especially in immunocompromised patients [16]. Recognizing the urgent need for new treatments, both the Centers for Disease Control and Prevention and the WHO have prioritized *A. baumannii* as a serious research target [17]. Global studies indicated that approximately 45% of all *A. baumannii* isolates are considered multidrug-resistant, with the ration reaching 90% in the Middle East, Turkey, and Greece [18].

Despite the efforts to develop a national action plan against antimicrobial resistance (AMR) in Lebanon, alarming rates were reported compared with global standards. A previous study conducted in Lebanese hospitals between 2011 and 2013 revealed a concerning prevalence of *A. baumannii*. The reported *Acinetobacter* spp susceptibility to piperacillin/tazobactam, cefepime, and imipenem was as low as 12.9%, 13.4%, and 17.6%, respectively [19]. The study also revealed that 17% of *A. baumannii* MDR was against colistin which is considered the last resort antibiotic against *A. baumannii*. In addition, a tertiary hospital in Lebanon reported a prevalence rate of 81% for carbapenem-resistant *A. baumannii* (CRAB) in 2018 [20]. A study conducted in a tertiary care hospital in Lebanon has revealed that the crude mortality rate associated with infections caused by *A. baumannii* was 63.5%, with 70.3% of the deaths attributed to bacteremia. The same study indicated that prolonged hospital stays, steroid use, and antibiotic exposure appeared to influence the mortality of *A. baumannii* bacteremia [21].

The absence of a robust national surveillance program reporting the incidence of MDR organisms in Lebanon presents a significant challenge in identifying the local prevalence of AMR specific to *A. baumannii*. Compounding this issue is the dearth of routine surveillance activities. Notably, certain regions and cities, including the capital of South Lebanon, Sidon, are often overlooked in studies. Furthermore, there is a notable scarcity of data concerning *A. baumannii*-associated mortality and its predictors. The emergence of resistance and the lack of novel antibiotics have led to the increased use of last-resort agents, which, if overprescribed, will further accelerate resistance rates. Therefore, to address this gap, our study aimed to (1) evaluate the antimicrobial susceptibility patterns of *A. baumannii*, (2) assess the appropriateness of the antimicrobial therapy administered to patients with *A. baumannii* infections, and (3) identify the mortality rate and its associated risk factors. This would provide valuable insight into the current landscape of *A. baumannii*, pave the way for proactive interventions, and generate population-specific data that are crucial for guiding the selection of empirical antimicrobial treatment in patients with nosocomial infections.

## Methods

### Study design and setting

This retrospective observational cross-sectional study was conducted among adult hospitalized patients (≥ 18 years old) who tested positive for *A. baumannii* isolates, over a 12-month period, from January 2021 to December 2021. The study was carried out at a university tertiary care hospital in Sidon, the capital of South Lebanon. This hospital is the largest in South Lebanon, with 325 beds, and admits approximately 14,000 patients annually.

### Inclusion and exclusion criteria

The medical record of all isolates that grew *A. baumannii* were reviewed. Inclusion criteria for the study included adult hospitalized patients (≥ 18 years old) who were infected with *A. baumannii.* Only the first episode of infection was retrieved for patients with multiple episodes. Patients with incomplete medical records, incomplete susceptibility testing profiles, pathogen colonization, poly-microbial infections, or those who were transferred out of the hospital within 48 h of infection acquisition were excluded. To exclude colonization, the review relied on documented clinical assessments and physicians' progress notes, explicitly classifying *A. baumannii* presence as colonization or infection. Patient-recorded signs and symptoms, and initiated targeted antibiotics were also considered. The exclusion of poly-microbial infections aimed to sharpen the focus on *A. baumannii* infections, ensuring the retrieval of specific information. For instance, it enables a detailed assessment of the appropriateness of the initial antibiotics received and its impact on treatment outcomes. This approach ensures a more homogeneous study population, reducing potential confounding factors associated with the presence of multiple pathogens.

Data were meticulously obtained through a thorough examination of patients' medical records subsequent to their discharge or post-mortem events. Given the absence of direct interaction with the enrolled patients, anonymized data were efficiently gathered utilizing a secure online database (REDCap, Vanderbilt University, Nashville, TN, USA).

### Antimicrobial susceptibility testing

Isolates were identified using biochemical gallery tests (API 20E; BioMérieux, Marcy l'Etoile, France). Antimicrobial susceptibility testing (AST), using Kirby-Bauer disk diffusion method, was conducted in accordance to the Clinical and Laboratory Standards Institute (CLSI) guidelines. The results were interpreted according to the cutoff values set by the CLSI document [22]. Plates were read manually using a ruler, followed by automatic reading using the ADAGIO system (Bio-Rad Laboratories, Hercules, CA, USA); an automated zone size reader for antimicrobial disk susceptibility tests. This system, functioning as both an imaging device and management software, automatically identifies antibiotic disks on the agar and measures the diameters of the surrounding inhibition zone [23]. The susceptibility of *A. baumannii* isolates to colistin was determined through the disk diffusion method, with a breakpoint set at a diameter of 11 mm.

### Data collection form development and structure

The data collection form was developed following an extensive literature review of relevant published studies [24–30]. The form comprised seven main sections consisting of closed-ended questions with pre-specified options. The first section aimed to gather patients’ socio-demographic information including age, sex, admission date, total duration of hospitalization, department of admission, site of infection acquisition, and the admitting diagnosis. In the second section, potential risk factors for mortality-associated infections were identified. These factors such included comorbid medical conditions, previous colonization, recent hospitalization within the past three months, antimicrobial use within the past three months, corticosteroid and immunosuppressant use, and invasive medical procedures performed prior to infection acquisition.

The third section focused on determining the primary site and the onset of infection along with the type of specimen collected. The fourth section was dedicated to identifying the empirical antibiotic therapy and assessing its appropriateness. This included the date of empirical treatment initiation in relation to the onset of infection, the specific antibiotic administered, the route of administration, dosage, and duration of therapy. Subsequently, the appropriateness of the received empirical therapy was evaluated based on prompt initiation, isolated pathogen susceptibility to the initiated antibiotics, adequate duration, proper dosage, and appropriate route of administration.

The fifth section obtained the antibiogram of the isolated pathogen including its susceptibility to various antimicrobial agents through laboratory testing. This information contributed to the classification of antimicrobial resistance specifically identifying whether the *A. baumannii* strain was Multi-Drug Resistant (MDR), Extensively Drug-Resistant (XDR), or Pan-Drug Resistant (PDR), as defined in the *Operational Definitions* section.

The sixth section focused on identifying whether empirical therapy was de-escalated after obtaining the culture results, along with recording the date of de-escalation. It also identified the specific antibiotic administered as definitive antimicrobial therapy, including its dose and route of administration. Finally, the seventh section identified treatment outcomes including the 30-day mortality following the onset of infection.

### Validity and reliability

The data collection form underwent a thorough review of face and content validity by an expert panel in the field. Three experts specialized in epidemiology, clinical pharmacy, and infectious diseases were selected to evaluate the clarity, understandability, and organization of the constructed form. To determine the content validity, the Item-Objective Congruence (IOC) index was applied. The experts were instructed to assign scores to each item based on the IOC scoring method, where 1 indicated that the expert was confident that the item measured the intended attribute, 0 indicated that the expert was uncertain about the item's ability to measure the attribute, and -1 indicated that the expert believed the item did not measure the attribute. The qualified items yielded an IOC score of 0.81, thus the form had acceptable content validity.

Moreover, a pilot test was conducted on 10 medical records using a convenience approach to assess the feasibility, practicality, suitability, and quality of the form. The data collected during the pilot test was included in the final analysis.

### Ethical considerations

The study was designed and conducted in accordance with the guidelines outlined in the World Medical Association Declaration of Helsinki [31]. The Institutional Review Board (IRB) of the assigned hospital granted approval for the study's protocol (No. #01-22). To safeguard patients' privacy and confidentiality, their medical records were anonymized and de-identified. The research team maintained no direct contact or follow-up with the patients. Given the retrospective observational nature of the study, where data collection occurred after patients' discharge or death, and given that no patient identifiers were accessed, the IRB waived the need for informed consent.

### Statistical analysis

Data were analyzed using the IBM Statistical Package for the Social Sciences (SPSS^®^) software version 24. The descriptive data were presented using frequencies and percentages for categorical variables, and mean with standard deviation for continuous variables. Continuous variables were tested for normality using Shapiro–Wilk tests before statistical comparisons. Time-to-mortality was analyzed using Kaplan–Meier survival analysis and log-rank test. Univariate analysis was performed to test the associations between mortality and independent categorical variables using Pearson’s chi-square test (χ^2^). Variables with *p*-value < 0.2 in the univariate analysis were considered for inclusion in logistic regression model in the multivariable analysis using a backward selection process. Adjusted odds ratio (AOR) was then calculated. Results with a *p*-value ≤ 0.05 and a 95% confidence interval were considered significant.

### Operational definitions

*Carbapenem-resistant A. baumannii (CRAB)*: refers to *A. baumannii* isolates that are resistant to either imipenem or meropenem [29].

*Carbapenem-susceptible A. baumannii (CSAB)*: refers to *A. baumannii* isolates that are sensitive to both imipenem and meropenem [29].

*Chronic kidney disease*: is defined as an estimated glomerular filtration rate (eGFR) of less than 60 mL/min/1.73 m^2^ for at least 3 months prior to admission [27].

*Extensively-drug resistant A. baumannii (XDRAB)*: refers to *A. baumannii* isolates that are only sensitive to colistin and/or tigecycline [28].

*Hospital-acquired infection*: also known as nosocomial infection, is defined as an infection in which onset of signs and symptoms occurs 48 h after hospital admission [32].

*Immunosuppressive therapy*: was defined as receipt of cytotoxic agents within 6 weeks, corticosteroids at a dosage ≥ 10 mg of prednisolone daily for more than 5 days within 4 weeks, or other immunosuppressive agents within 2 weeks prior to the onset of infection [27].

*Initial appropriate antimicrobial therapy*: is defined as the administration ≥ 1 antimicrobial agents, to which the causative pathogen was susceptible according to the susceptibility tests, within 48 h of onset of infection. The therapy should be administered using an approved route and dosage regimen appropriate for end-organ function. Antimicrobial therapy that does not meet this definition is considered inappropriate [27].

*Length of stay (LOS) prior to event*: refers to the duration of hospitalization before the onset of infection [33].

*Multi-drug resistant A. baumannii (MDRAB)*: refers to the resistance of *A. baumannii* to three or more classes of antimicrobials, including antipseudomonal cephalosporins, antipseudomonal carbapenems, ampicillin-sulbactam, fluoroquinolones, and aminoglycosides [28].

*Non-XDR*: includes non-multidrug resistant (non-MDR) and multidrug resistant (MDR) *A. baumannii* isolates.

*Onset of infection*: defined as the day when the culture, which eventually grew *A. baumannii*, was obtained [26].

*Pandrug resistant A. baumannii (PDRAB)*: refers to *A. baumannii* isolates that demonstrate resistance to all available antibiotics [28].

*Previous antibiotic therapy*: is defined as receiving any systemic antibiotic for a minimum of 72 h within a 3-month period prior to the onset of infection [27].

*Recent surgery*: is defined as a medical operation performed within 4 weeks prior to the onset of infection [27].

*The main outcome of the study*: is the all-cause 30-day mortality following the onset of infection [30].

*Time-to-mortality*: is defined as the duration between the onset of infection and the occurrence of death [30].

## Results

### Screened and enrolled cases

**Fig. 1:**
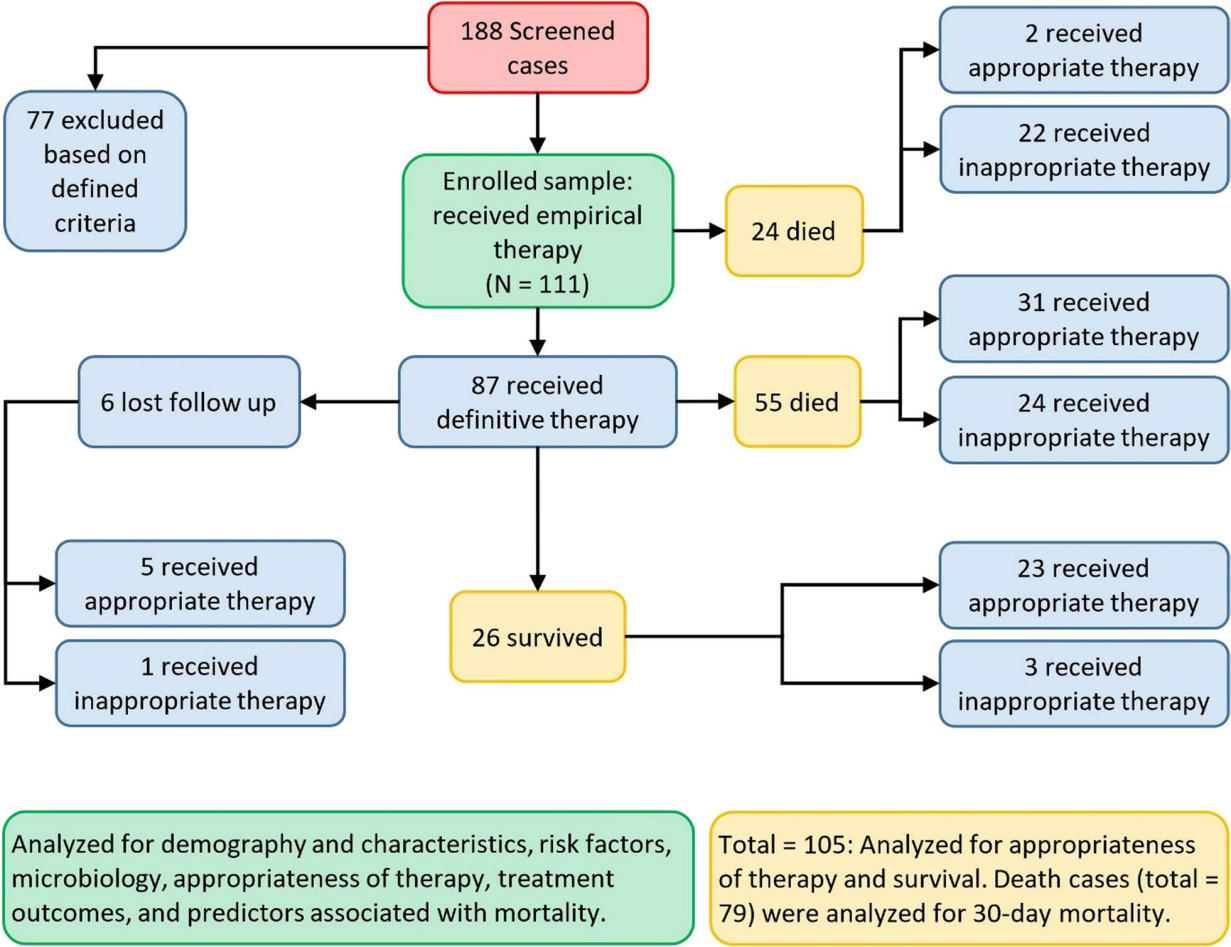
Selection, inclusion, and exclusion of cases of *A. baumannii* infection, and relevant analysis

The total number of screened cases with positive *A. baumannii* bacteriologic specimens was 188. Of these, 77 cases were excluded based on the following criteria: patients < 18 years (n = 14), patients with community-acquired *A. baumannii* infection (n = 19), patients with poly-microbial infections (n = 24), patients ‏discharged within 48 h of infection acquisition (n = 8), and patients with ‏incomplete medical records (n = 12). Hence a total of 111 patients with *‏A. baumannii* infection were included in this study. However, only 105 cases were included in the final analysis of the treatment outcome and the 30-day mortality because six patients failed to show up during the follow-up period (Fig. 1).

### Patient characteristics

Table 1 shows the characteristics and risk factors for mortality associated with *A. baumannii* infection for the 111 included patients, whose ages ranged from 23 to 97 years with around 62% of them being males. Most infections (≈ 77%) were acquired in the ICU and the highest rate of infection (≈ 44%) was reported during the winter. Most patients (105, 94.6%) were suffering from at least one underlying medical condition with hypertension being the most common of which (≈ 65%). Previous hospitalization, in addition to the use of antibiotics, glucocorticoids, and chemotherapy before the *A. baumannii* infection were reported by around 11%, 13%, 12%, and 9% of the cases, respectively.

**Table 1:** Patient characteristics and potential risk factors for mortality associated with *A. baumannii* infections (N = 111)

Information	n (%)
*Sex*	
Male	69 (62.2)
Female	42 (37.8)
*Age (years)*	
< 45	10 (9)
45–54	6 (5.4)
55–64	27 (24.3)
65–74	20 (18)
≥ 75	48 (43.2)
Mean ± standard deviation: 68 ± 16	
Range: 23–97	
*Department of infection acquisition*	
Intensive care unit	85 (76.6)
Internal medicine	13 (11.7)
Cardiac care unit	9 (8.1)
Surgery	3 (2.7)
Oncology	1 (0.9)
*Season of infection acquisition*	
Winter	49 (44.1)
Spring	18 (16.2)
Summer	31 (27.9)
Fall	13 (11.7)
Potential pre-admission risk factors	
*Underlying medical conditions*^*a*^	
Hypertension	72 (64.9)
Diabetes mellitus type II	63 (56.8)
Chronic kidney disease	37 (33.3)
Coronary artery disease	37 (33.3)
Neutropenia	33 (29.7)
Thrombocytopenia	30 (27)
Solid neoplasia	26 (23.4)
Dyslipidemia	22 (19.8)
Blood transfusion	22 (19.8)
Chronic obstructive lung disease	13 (11.7)
Cerebrovascular disease	12 (10.8)
Dementia	12 (10.8)
Congestive heart failure	10 (9)
Peripheral vascular disease	7 (6.3)
Epilepsy	3 (2.7)
Lymphoma	3 (2.7)
Leukemia	3 (2.7)
Peptic ulcer	3 (2.7)
Polytrauma	2 (1.8)
Solid organ transplant	1 (0.9)
HIV/AIDS	1 (0.9)
*Hospitalization within the last three months*	12 (10.8)
*Use of antibiotics within the last three months*	14 (12.6)
*Use of glucocorticoids within the last four weeks*^*b*^	13 (11.7)
*Use of chemotherapy within the last three months*	10 (9.1%)
Potential post-admission risk factors	
*Length of hospital stay prior to infection acquisition (days)*	
Mean ± standard deviation: 12.23 ± 8.25	
*Invasive medical procedures within 30 days prior to infection acquisition*^*a*^	
Mechanical ventilation	78 (70.3)
Indwelling urethral catheterization	73 (65.8)
Central venous line	58 (52.3)
Surgery	23 (20.7)
Prolonged total parenteral nutrition	23 (20.7)
Renal replacement therapy (dialysis)	20 (18)

^a^As multiple responses were recorded from certain patients, numbers do not add up to 111

^b^≥ 10 mg of prednisolone daily for > 5 days within 4 weeks prior to the onset of infection (or an equivalent glucocorticoid and dosage)

The mean duration of hospitalization, including the period prior to the infection acquisition (12.23 ± 8.25), was 22.4 ± 16.3 days, ranging from 5 to 47 days. Around 94% of patients underwent invasive medical procedures within 30 days prior to infection acquisition, where mechanical ventilation was the highest reported procedure (≈ 70%).

### Microbiological characteristics of the *A. baumannii* isolates

The *A. baumannii* isolates were derived from specimens of deep tracheal aspirates (57, 51.4%), sputum (27, 24.3%), skin wound/exudates (15, 13.5%), blood (11, 9.9%), and urine (1, 0.9%). The main source of infection was the respiratory tract (89, 80.2%), followed by the soft and skin tissues (15, 13.5%), the bloodstream (6, 5.4%), and the urinary tract (1, 0.9%).

‏All isolates were sensitive to colistin and tigecycline while most of them (≈ 96%) were resistant to ampicillin/sulbactam and aztreonam, as seen in Table 2. Among the 111 cases, 59 (53.2%) were multidrug resistant (MDR), 48 (43.2%) were extensively drug resistant (XDR), and only 4 cases (3.6%) were non-MDR. Notably, none of the cases were pan-drug resistant (PDR). However, most cases (105, 94.6%) were carbapenem-resistant *A. baumannii* (CRAB).

**Table 2:** In vitro antimicrobial susceptibility results for *A. baumannii* isolates (N = 111)

Antibiotics	Isolates (n)	Resistant n (%)	Sensitive n (%)	Intermediate n (%)
Colistin	111	0 (0)	111 (100)	0 (0)
Tigecycline	111	0 (0)	111 (100)	0 (0)
Trimethoprim/sulfamethoxazole	111	52 (46.8)	57 (51.4)	2 (1.8)
Minocycline	111	97 (87.4)	10 (9)	4 (3.6)
Gentamicin	110^*^	100 (90.9)	10 (9.1)	0 (0)
Ciprofloxacin	111	104 (93.7)	7 (6.3)	0 (0)
Piperacillin/tazobactam	110^*^	104 (94.5)	6 (5.5)	0 (0)
Amikacin	111	105 (94.6)	6 (5.4)	0 (0)
Cefepime	111	105 (94.6)	6 (5.4)	0 (0)
Imipenem/cilastatin	111	105 (94.6)	6 (5.4)	0 (0)
Meropenem	111	105 (94.6)	6 (5.4)	0 (0)
Ceftazidime	111	106 (95.5)	4 (3.6)	1 (0.9)
Doxycycline	111	106 (96.4)	4 (3.6)	0 (0)
Ampicillin/sulbactam	111	107 (96.4)	4 (3.6)	0 (0)
Aztreonam	111	107 (96.4)	4 (3.6)	0 (0)

*A report was missing

### Assessment of antimicrobial therapy appropriateness

Out of the 111 included patients, 47 (42.3%) received a combination of empirical antimicrobial therapy while the rest were initiated on monotherapy as seen in Table 3. Meropenem was the most received antibiotic (64, 57.6%) while ceftazidime was the least (5, 4.5%). Almost half of the patients (n = 50, 45.1%) out of the total 111 received initially inappropriate antimicrobial therapy. Among them, 6 patients (12% of the 50) experienced a delay in receiving the initial antibiotic therapy, administered after 48 h of the onset of infection. Additionally, 46 patients (92% of the 50) were initially prescribed antibiotics to which the causative pathogen was resistant based on susceptibility tests. Furthermore, 3 patients (6% of the 50) received initial antibiotics with an inappropriate dosage regimen. However, all patients received the initial antimicrobial therapy with appropriate route of administration.

**Table 3:** Empirical and definitive antimicrobial therapy received

Antimicrobial therapy	n (%)
Empirical antimicrobial therapy (N = 111)^a^	
*Monotherapy vs combination therapy*	
Monotherapy	64 (57.6)
Combination	47 (42.3)
*Empirical antimicrobial therapy initiated*	
Meropenem	64 (57.6)
Colistin	59 (53.1)
Vancomycin	27 (24.3)
Piperacillin/tazobactam	15 (13.5)
Levofloxacin	13 (11.7)
Teicoplanin	10 (9.2)
Ceftazidime	5 (4.5)
Others^b^	7 (6.3)
*Definitive antimicrobial therapy (N = 87)*^c^	
Colistin	80 (92)
Meropenem	4 (4.6)
Tigecycline	3 (3.4)

^a^As multiple responses were recorded, numbers do not add up to 111

^b^Cefepime, amikacin, ciprofloxacin, imipenem/cilastatin, ceftriaxone, clindamycin, and ampicillin/sulbactam

^c^Twenty-four patients out of the 111 died during the empirical therapy

It is noteworthy that 24 patients (21.8%) died while receiving the empirical antimicrobial therapy. The survived 87 patients were shifted to definitive antibiotic therapy, where most of them (80, 92%) were placed on colistin.

### Infection prognosis and treatment outcomes

The following was observed among the 111 enrolled cases: sepsis/septic shock (61, 55%), renal failure (66, 59.5%), and/or acute respiratory distress syndrome (35, 31.5%). Unfortunately, 79 patients (71.2%) died within 30 days after infection acquisition (including the 24 who died during the empirical therapy).

### Mortality associated with *A. baumannii* infection

**Fig. 2:**
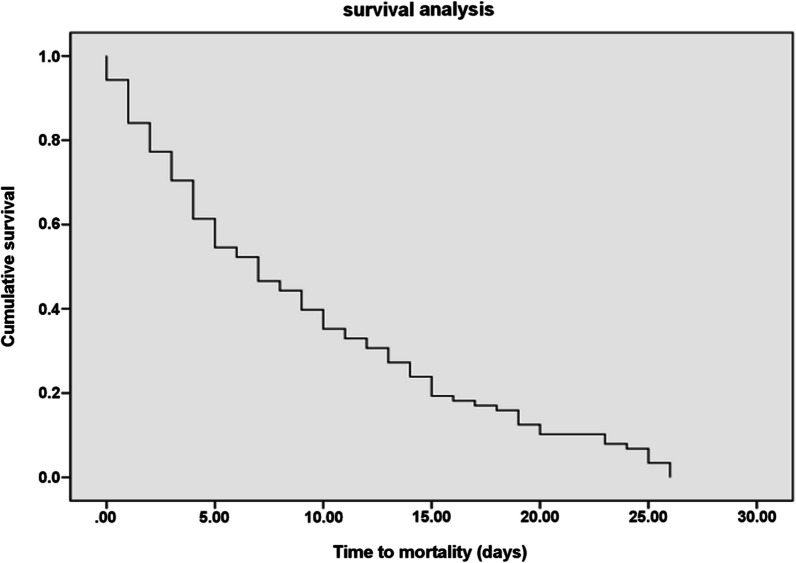
The Kaplan–Meier survival curve for patients with A. baumannii infection

Six patients (out of the enrolled 111) failed to show up during the follow-up period since they left the hospital despite the medical advice. The 30-day, all-cause mortality rate of *A. baumannii* infection among the remaining 105 cases was 75.2% (79 patients). The mean time-to-mortality was 9.06 ± 7.66 days (Fig. 2).

### Comparison of survival of patients with *A. baumannii* infections according to the appropriateness of antimicrobial therapy

**Fig. 3:**
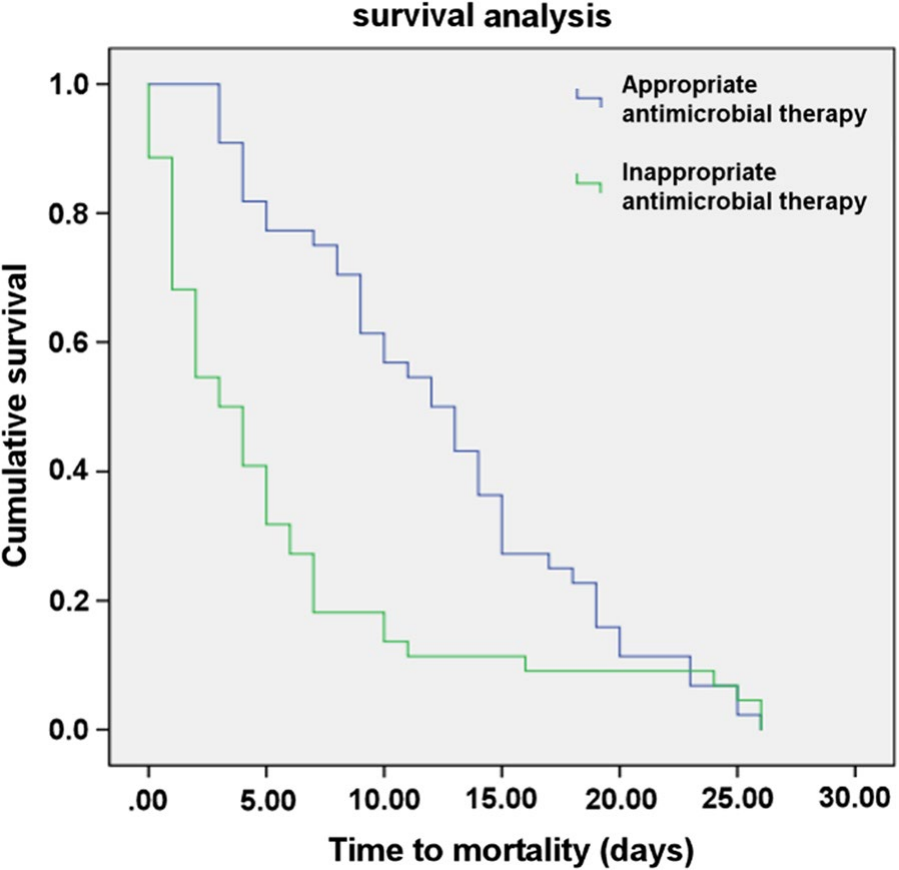
The Kaplan–Meier survival curves for patients with *A. baumannii* infection according to the appropriateness of the received antimicrobial therapy

As shown in Fig. 3, the Kaplan–Meier survival curve in *A. baumannii* infections revealed a significantly shorter time to mortality in patients who died after receiving inappropriate antimicrobial therapy than those who died after receiving appropriate antimicrobial therapy (46 and 33 out of 79, respectively, log-rank test = 11.49, *P* = 0.001). The mean time to death for patients who received inappropriate antimicrobial therapy was 5.70 ± 1.07 days, whereas the mean time to death for patients who ‏received appropriate antimicrobial ‏therapy ‏was 12.43 ± 1.01 days (Table 4).

**Table 4:** Comparison of survival time between patients who died after receiving appropriate antimicrobial therapy and those who died after receiving inappropriate antimicrobial therapy

Group	30-days mortality	Mean survival time (days)	95% CI	Log-rank *X*^2^ test	*P*
	n (%)	Mean ± SD			
Appropriate antimicrobial therapy (N = 56)	33 (41.8)	12.43 ± 1.01	10.45 ± 14.14	11.49	0.001*
Inappropriate antimicrobial therapy (N = 49)	46 (58.2)	5.70 ± 1.07	3.6 ± 7.80		

CI, Confidence interval; SD, standard deviation

*Statistically significant (*P* < 0.05)

### Comparison of survival between patients who died due to infection with XDR versus non-XDR *A. baumannii* isolates

**Fig. 4:**
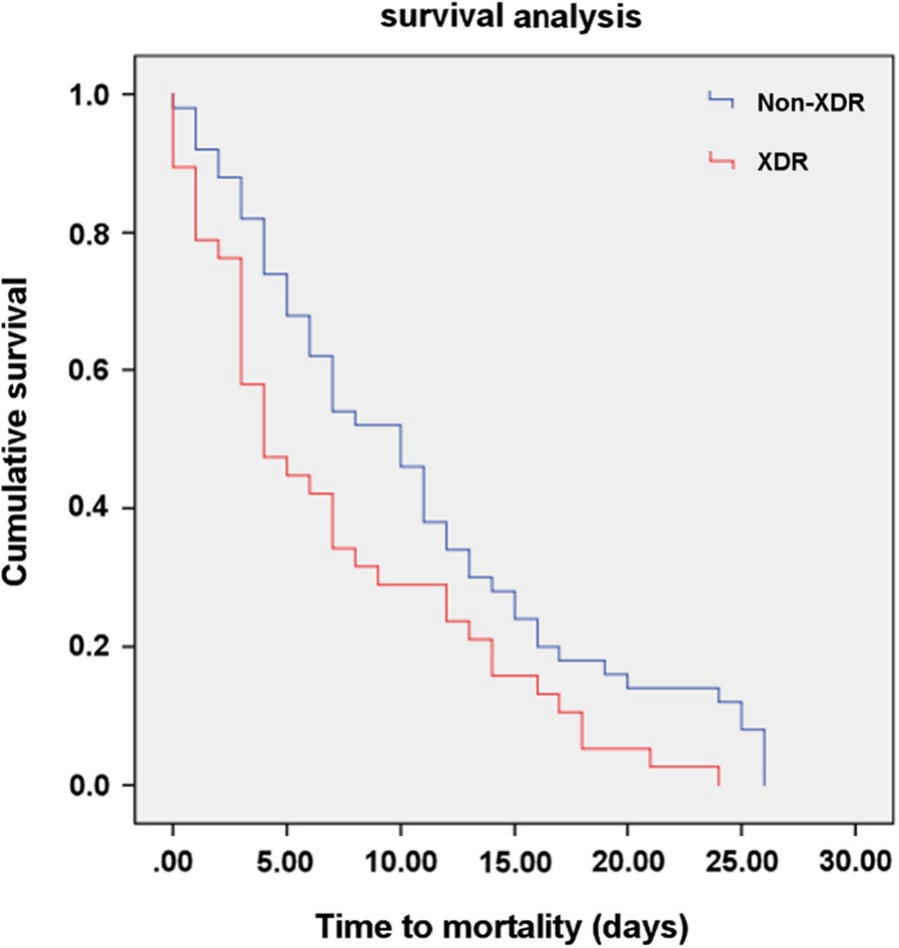
The Kaplan–Meier survival curves for patients with extensively drug resistant (XDR) and non-XDR *A. baumannii* infection

As mentioned above, 79 patients died within 30 days of acquiring the infection. The survival curve revealed a significantly shorter time to mortality in patients with XDR than those with non-XDR (includes non-multidrug resistant and multidrug resistant *A. baumannii*) isolates (log-rank test = 5.45, *P* = 0.002) (Fig. 4). The mean time to death in the former group was 7.2 ± 1.0 days compared with 10.7 ± 1.1 days in the latter group (Table 5).

**Table 5:** Comparison of survival time between patients infected with XDR and non-XDR *A. baumannii* isolates

Group	30-days mortality	Mean survival time (days)	95% CI	Log-rank *X*^2^ test	*P*
	n (%)	Mean ± SD			
Non-XDR^a^ (N = 61)	41 (51.8)	10.7 ± 1.1	8.5 ± 12.8	5.45	0.02*
XDR (N = 44)	38 (48.1)	7.2 ± 1.0	5.0 ± 9.2		

CI, Confidence interval; SD, standard deviation; XDR, extensively drug resistant

^a^Includes non-multidrug resistant and multidrug resistant *A. baumannii* isolates

*Statistically significant (*P* < 0.05)

### Predictors of mortality in patients with *A. baumannii* infection

The univariate analysis has identified a significant association between antimicrobial resistance and mortality. XDR isolates were found to be significantly associated with a higher mortality rate compared with non-MDR and MDR isolates (unadjusted odds ratio [UOR] = 3.09, 95% CI 1.12–8.51, *P* = 0.02) (Table 6). Binary logistic regression, using backward stepwise analysis, revealed three predictors that were significantly associated with mortality in patients with *A. baumannii* infection (Table 7). Accordingly, inappropriate antimicrobial therapy (adjusted odds ratio [AOR] = 16.22, 95% CI 2.68–9.97, *P* = 0.002), mechanical ventilation (AOR = 14.72, 95% CI 3.27–6.61, *P* < 0.001) and ‏thrombocytopenia (AOR = 8.82, 95% CI 1.12–9.75, *P* = 0.03) were more likely associated with mortality. However, the length of hospital stay prior to acquiring the infection was insignificantly related to mortality as revealed by Mann–Whitney U test (*P* = 0.06).

**Table 6:** Univariate analysis of predictors associated with mortality in patients with *A. baumannii* infection (N = 105)^a^

Characteristics	n (%)^b^	30-Days all-cause mortality		UOR (95% CI)	Pearson’s *X*^2^	*P*^d^
		Survived (26) n (%)^c^	Died (79) n (%)^c^			
Age group (reference: 55–64 years)						
< 45	9 (8.6)	5 (55.6)	4 (44.4)	0.45 (0.10–2.12)	10.40	0.04^e,f^
45–54	5 (4.8)	0 (0)	5 (100)	–		
55–64	25 (23.8)	9 (36)	16 (64)	–		
65–74	19 (18.1)	5 (26.3)	14 (73.7)	1.58 (0.43–5.82)		
≥ 75	47 (44.8)	7 (14.9)	40 (85.1)	3.21 (1.05–10.10)		
Sex (reference: female)						
Male	63 (60)	15 (23.8)	48 (76.2)	1.14 (0.46–2.79)	0.07	0.47
Female	42 (40)	11 (26.2)	31 (73.8)	–		
Department of infection acquisition (reference: internal medicine)						
Intensive care unit	83 (79)	11 (13.3)	72 (86.7)	8.18 (1.90–35.23)	33.26	< 0.001^e,g^
Internal medicine	9 (8.6)	5 (55.6)	4 (44.4)	–		
Cardiac care unit	10 (9.5)	8 (80)	2 (20)	–		
Surgery	1 (1)	0 (0)	1 (100)	–		
Oncology	2 (1.9)	2 (100)	0 (0)	–		
Past medical history (reference: no history of the related condition)						
Hypertension	68 (64.8)	16 (23.5)	52 (76.5)	1.20 (0.48–3.01)	0.157	0.69
Diabetes mellitus	61 (58.1)	16 (26.2)	45 (73.8)	0.83 (0.33–2.05)	0.168	0.68
Neutropenia	32 (30.5)	5 (15.6)	27 (84.4)	2.18 (0.74–6.42)	2.06	0.15
Thrombocytopenia	30 (28.6)	2 (6.7)	28 (93.3)	6.59 (1.45–29.95)	7.38	< 0.001^e^
Chronic kidney disease	36 (34.3)	6 (16.7)	30 (83.3)	2.04 (0.74–5.66)	1.92	0.17
Coronary artery disease	33 (31.4)	9 (27.3)	24 (72.7)	0.82 (0.32–2.11)	0.163	0.69
Previous hospitalization within the last 3 months (reference: no)						
No	94 (89.5)	23 (24.5)	71 (75.5)	–	0.042	0.83
Yes	11 (10.5)	3 (27.3)	8 (72.7)	0.86 (0.21–3.53)		
Previous antibiotics use within the last 3 months (reference: no)						
No	93 (88.6)	24 (25.8)	69 (74.2)	–	0.477	0.49
Yes	12 (11.4)	2 (16.7)	10 (83.3)	1.74 (0.36–8.51)		
Invasive procedures within 30 days prior to infection acquisition (reference: no)						
No	7 (6.7)	4 (57.1)	3 (42.9)	–	4.22	0.04^e^
Yes	98 (93.3)	22 (22.4)	76 (77.6)	4.61 (0.96–22.15)		
Mechanical ventilation (reference: no)						
No	28 (26.7)	18 (64.3)	10 (35.7)	–	32.01	< 0.001^e^
Yes	77 (73.3)	8 (10.4)	69 (89.6)	15.53 (5.35–45.02)		
Indwelling urethral catheterization (reference: no)						
No	32 (30.5)	9 (28.1)	23 (71.9)	–	0.27	0.59
Yes	73 (69.5)	17 (23.3)	56 (76.7)	1.29 (0.5–3.31)		
Central venous line (reference: no)						
No	47 (44.8)	19 (40.4)	28 (59.6)	–	11.20	0.001^e^
Yes	58 (55.2)	7 (12.1)	51 (87.9)	4.94 (1.85–13.19)		
Major surgery (reference: no)						
No	86 (81.9)	14 (16.3)	72 (83.7)	–	18.35	0.22
Yes	19 (18.1)	12 (63.2)	7 (36.8)	0.11 (0.04–0.34)		
Prolonged total parenteral nutrition (reference: no)						
No	82 (78.1)	22 (26.8)	60 (73.2)	–	0.85	0.35
Yes	23 (21.9)	4 (17.4)	19 (82.6)	1.74 (0.53–5.69)		
Renal replacement therapy (dialysis) (reference: no)						
No	85 (81)	24 (28.2)	61 (71.8)	–	2.89	0.08
Yes	20 (19)	2 (10)	18 (90)	3.54 (0.76–16.44)		
Source of infection (reference: soft tissue/wound infection)						
Respiratory infection	86 (81.9)	14 (16.3)	72 (83.7)	8.23 (2.34–28.88)	19.13	< 0.001^e^
Soft tissue/wound infection	13 (12.4)	8 (61.5)	5 (38.5)	–		
Blood stream infection	5 (4.8)	3 (60)	2 (40)	1.07 (0.13–8.79)		
Urinary tract infection	1 (1)	0 (0)	1 (100)	–		
Type of antimicrobial resistance (references: non-XDR and CSAB)						
*Non-XDR versus XDR*						
Non-XDR^h^	61 (58.1)	20 (32.8)	41 (67.2)	–	5.03	0.02^e^
XDR	44 (41.9)	6 (13.6)	38 (86.4)	3.09 (1.12–8.51)		
*CSAB versus CRAB*						
CSAB	6 (5.7)	6 (100)	0 (0)	–	19.33	< 0.001^e^
CRAB	99 (94.3)	20 (20.2)	79 (79.8)	–		
Appropriateness of the antimicrobial therapy (reference: appropriate)						
Appropriate	56 (53.3)	23 (41.1)	33 (58.9)	–	17.13	< 0.001^e^
Inappropriate	49 (46.7)	3 (6.1)	46 (93.9)	10.69 (2.96–38.57)		

CRAB, Carbapenem-resistance *Acinetobacter baumannii*; CSAB, Carbapenem-sensitive *Acinetobacter baumannii*; SD; standard deviation; UOR, unadjusted odds ratio; XDR, extensively drug resistant

^a^Six patients (out of the 111) who failed to show up during the follow-up period were excluded

^b^Percentages for the column

^c^Percentages for the row

^d^Univariate analysis was conducted to test the associations between variables with mortality

^e^Statistically significant (*P* < 0.05)

^f^The only significant difference was observed between the age group ≥ 75 and all other groups

^g^The only significant difference was observed between the intensive care unit and all other groups

^h^Includes non-multidrug resistant and multidrug resistant *A. baumannii* isolates

**Table 7:** Logistic regression analysis^a^ of the significant predictors associated with mortality in patients with *A. baumannii* infection

Predictors	UOR	B	SE	Wald	AOR	95% CI	*P*
Constant		− 1.735	0.70	6.07	0.17		0.01
Appropriateness of the antimicrobial therapy (reference: appropriate)							
Inappropriate	10.69	2.78	0.91	9.22	16.22	2.68–9.97	0.002^b^
Mechanical ventilation (reference: no)							
Yes	15.53	2.68	0.76	12.29	14.72	3.27–6.61	< 0.001^b^
Thrombocytopenia (reference: no)							
Yes	6.59	2.34	1.13	4.25	8.82	1.12–9.75	0.03^b^

AOR, adjusted odds ratio; B, coefficient for the constant in the null model; CI, confidence interval; SE, standard error; UOR, unadjusted odds ratio; Wald, Wald chi-square test that tests the null hypothesis

^a^Binary logistic regression using backward stepwise analysis

^b^Statistically significant (*P* < 0.05)

## Discussion

The current study has revealed that the main site of *A. baumannii* infections was the lower respiratory tract. Similarly, a seven-year prospective cohort study between 2007 and 2014 in a tertiary care hospital in Lebanon revealed that the common *A. baumannii* infections consisted of respiratory infections (53.1%) [34]. In addition, the *A. baumannii* isolates obtained in this study were mainly MDR and XDR, with no recorded PDR isolates. This high antimicrobial resistance owes to the high levels of *A. baumannii*’s intrinsic resistance to antibiotics and its ability to easily acquire new resistance determinants, thus limiting treatment options for infected patients [35].

We found that 95% of *A. baumannii* strains obtained in this study were resistant to carbapenems (CRAB). Previous studies have shown that the incidence of CRAB in Lebanon ranged from 58 to 100% [20, 36–40]. The observed variation in CRAB rates across national studies might be attributed to potential methodological differences, geographical influences, patient population characteristics, discrepancies in institutional antibiotics prescribing practices, and fluctuations in resistance patterns over time. Therefore, standardizing surveillance methodologies and emphasizing consistent data reporting practices are essential for a comprehensive understanding and effective management of the complex factors contributing to CRAB resistance patterns at a national level. Jordan and Iraq, near Levant countries with low-to-middle income, recorded high rates of CRAB isolates ranging from 84 to 99.2% [41–43]. This is congruent with the results of a multinational clinical trial conducted among three European countries (Greece, Spain, and Italy), where 97% of the isolates (all except two) were resistant to imipenem [35]. CRAB has been reported worldwide and has become a serious health problem due to the limited treatment options [44, 45]. Although carbapenem consumption has been linked to an increase in *A. baumannii* resistance rates [46], mathematical models, however, have outlined the possible impact of reducing carbapenem consumption on resistance acquisition among microbes, including CRAB [47]. A study at a Lebanese tertiary care hospital examined the genetic relatedness of CRAB strains amid a suspected outbreak, utilizing real-time polymerase chain reaction (PCR), pulsed-field gel electrophoresis, and multilocus sequence typing analyses. The investigation revealed multiple clones with blaOXA-23, with one also carrying blaNDM-1. Despite the endemic presence of various clones, the hospital effectively implemented infection prevention and control measures, resulting in the eradication of the outbreak of OXA-23 producing ST2 CRAB [40].

Lately, colistin is considered the main and salvage therapy for nosocomial infections caused by CRAB [48, 49]. Fortunately, all isolates obtained in this study were susceptible to colistin. In support of our findings, a recent review article that identified 40 studies investigating the incidence of antimicrobial resistance in Lebanon, within a 5 years’ interval from 2015 to 2020, reported that the overall *A. baumannii* susceptibility to colistin was 99–100% [50]. However, an earlier nationwide surveillance of antimicrobial resistance conducted among 16 Lebanese hospitals between 2011 and 2013, reported that *A. baumannii* resistance to colistin was 17% [19]. This discrepancy reflects the trend of *A. baumannii* resistance indicating a decreased rate of resistance to colistin. Conversely, a recent ‏bibliometric analysis investigating the global trends of colistin resistance reported a significant growth of colistin ‏resistance in the past decade [51]. There is an urgent need for implementing a ‏perspicacious strategy to contain this threat because colistin is the last line of defense and because the ‏emergence of colistin resistance will head us to a post-antibiotic era, in which common ‏infections might be lethal. It is essential to note that the rate of resistance to colistin in Lebanon cannot be directly compared with international resistance rate, as the observed variation may be attributed to the utilization of the disk diffusion test instead of the minimum inhibitory concentration (MIC) determination via broth dilution, as recommended by the Clinical and Laboratory Standards Institute (CLSI) and the European Committee on Antimicrobial Susceptibility Testing (EUCAST). The use of disk diffusion test for colistin may not yield as accurate results as the MIC determination method, particularly for colistin. Therefore, adopting the EUCAST and CLSI-recommended MIC determination method is crucial to ensure more accurate, reliable, and comparable results in assessing colistin susceptibility [23, 52, 53].

Controlling the spread of *A. baumannii* resistance in Lebanon requires a multifaceted approach. Numerous evidence-based interventions have been established to address the ubiquitous threat of MDRAB infections. Antimicrobial stewardship programs (ASPs) have been established to control the misuse and overuse of antibiotics, which can limit the pressure that drives the emergence and spread of *A. baumannii* resistant strains [54, 55]. The ASPs have resulted in noticeable decreases in CRAB infection rates [54, 55]. To prevent the wide dissemination of MDRAB among healthcare personnel and patients, the implementation of strict infection control protocols is essential. However, in Lebanon, this is challenging due to staff shortages [54]. One approach to limit transmission is through staff cohorting, which involves assigning specific healthcare personnel to care only for patients infected or colonized with a single target pathogen [56].

In 2019, the Lebanese Ministry of Public Health, with the assistance of the WHO, organized a national action plan to tackle MDR organisms aiming to optimize antibiotic use by implementing ASPs [55]. However, additional initiatives are required to achieve effective results. For instance, a study conducted at a tertiary care center in Lebanon between 2018 and 2020 demonstrated that the implementation of ASPs with carbapenem-sparing strategies and infection control interventions resulted in a significant drop in CRAB resistance rates from 82% in 2019 to 63% in 2020 [57]. These findings highlight the potential benefits of replicating such interventions in other hospitals struggling with high rates of CRAB infections.

This study also revealed an alarmingly high mortality rate of 72%. Our finding is consistent with the mortality rate reported by other national studies, which ranged from 52 to 70% [34, 58]. However, the worldwide mortality rate of patients with *A. baumannii* infection ranged between 26 and 60% [59–65]. The higher mortality rate in Lebanon, compared to other countries, could be attributed to several factors. The current study and other national studies have detected all-cause mortality, rather than mortality specifically attributed to the infection. Other possible contributing factors to the high mortality rate include the patient’s underlying medical illness, antimicrobial resistance, and treatment appropriateness.

Numerous risk factors have been investigated as potential predictors of mortality in patients infected with *A. baumannii*. This study revealed that antimicrobial resistance, inappropriate antimicrobial treatment, thrombocytopenia, and invasive mechanical ventilation are significantly associated with a higher mortality rate. The univariate analysis showed that the XDR isolates were significantly associated with higher mortality compared to non-MDR and MDR isolates. Nevertheless, the association between mortality and drug resistance in *A. baumannii* infections is still controversial [66, 67]. While some studies suggest that resistance to antimicrobials by *A. baumannii* may lower survival rates due to its enhanced virulence or delays in initiating effective treatments, others have reported conflicting findings [68–70].

The current study documented a high rate of inappropriate initiation of antimicrobial therapy, which was significantly associated with increased mortality rates. This finding is consistent with previous studies that have shown the negative impact of inappropriate antimicrobial therapy use in *A. baumannii* infections on patient survival. This becomes more evident with patients who receive inappropriate empirical antimicrobial therapy resulting in higher mortality rate compared to those who receive appropriate treatment [59, 70, 71]. Therefore, early initiation of appropriate antibiotic therapy is crucial for saving lives. To ensure optimal patient outcomes, healthcare providers should judiciously choose antibiotics for nosocomial infections, considering factors such as patient’s clinical manifestation, source of infection, commonly associated MDR organisms, severity of illness, patient’s risk factors, and local resistance patterns. Annual surveillance is necessary to monitor *A. baumannii* infections and identify the new resistance patterns, which can help guide treatment decisions and identify trends of resistance. Additionally, it is important to consider the “five rights” when administering antimicrobial therapy to patients, which include the right patient, right medication, right dose, right route of administration, and right time. Moreover, it is recommended to implement appropriate de-escalation upon receipt of culture results [72].

The appropriate treatment of MDRAB remains a topic of debate despite its proven impact on mortality rates. Colistin and tigecycline are the only available options for *A. baumannii* treatment, despite of their nephrotoxic and neurotoxic profiles [73]. Limited evidence exists regarding the efficacy of combination therapy versus monotherapy, and there is a lack of a clear standard antibiotic regimen for the treatment of CRAB infections due to inadequate comparative effectiveness studies [73, 74]. A single active agent may be sufficient for mild CRAB infections but combination therapy with at least two agents is suggested for moderate to severe infections due to limited clinical data on the effectiveness of any single antibiotic agent [74].

Although a retrospective cohort study conducted between 2015 and 2017 showed that a combined treatment of colistin plus meropenem had better clinical and microbiological responses [75], a randomized controlled trial in 2018 argued against the superiority of combination therapy [76]. Currently, intravenous colistin is the only remaining therapeutic option while there is no solid evidence on the use of inhaled colistin in the management of pneumonia [77]. Tigecycline is recommended for the treatment of CRAB but its use is limited, particularly after the FDA issued a black box warning due to its association with higher mortality in ventilator-associated pneumonia (VAP) [78]. This was supported by the guidelines of the Infectious Diseases Society of America and the American Thoracic Society in 2016, which were against the use of tigecycline due to its decreased therapeutic efficacy in VAP and increased mortality rates compared with colistin-containing regimens [79].

Mechanical ventilation has been found to be strongly associated with mortality in patients infected with *A. baumannii*. This finding is consistent with several studies that have identified mechanical ventilation as a significant predictor of *A. baumannii*-related mortality [80–82]. For instance, a retrospective study conducted in a China reported that patients infected with *A. baumannii* who received invasive mechanical ventilation had a ninefold increase in the odds of mortality [82]. There are several possible explanations for this association including the severity of illness, the need for mechanical ventilation, cross-transmission and invasiveness of pathogens, and the potential complications associated with ventilation, which can compromise the host’s normal defense barriers and potentially affect the disease prognosis [80–82].

Thrombocytopenia has also been identified as a significant predictor associated with mortality in patients infected with *A. baumannii*. Consonantly, several studies have reported a significant association between thrombocytopenia and mortality in infected patients [81, 83, 84]. A recent six-year study conducted in China found that thrombocyte count < 50 × 10^9^/L has increased the risk of 30-day mortality in infected patients by 9-folds (AOR = 8.72, 95% CI 1.93–39.3, *P* < 0.001) [85]. Similarly, a retrospective study in an Indian tertiary hospital also reported a significant association between low platelets of less than 1.5 lacs/mm^3^ and mortality in patients with *A. baumannii* bacteremia (AOR = 3.77, 95% CI 0.85–16.57, *P* = 0.04) [83]. Thrombocytopenia indicates the severity of the infection and suggests a poor prognosis as low platelet counts weaken the patient’s immunity, hinder pathogen capture, and inhibit inflammatory cytokines [84]. Nonetheless, the exact association of thrombocytopenia with mortality is not fully understood. It is thought that thrombocytopenia may be a marker of severe illness and systemic inflammation and may contribute to the development of sepsis, disseminated intravascular coagulation, and multi-organ damage [81, 83–85]. Thus‏, ‏it is essential to monitor platelet counts in infected patients to identify disease prognosis and ‏provide appropriate management.

### Study limitations

This study has several limitations that should be noted. First, the study’s retrospective nature, coupled with the absence of a control group, has constrained our capacity to control potential confounding variables and hindered the verification of predictors of mortality attributed to *A. baumannii* infections. Second, the study’s single-center design and relatively small sample size may limit the generalizability of the findings, potentially not reflecting the practices and susceptibility patterns of *A. baumannii* in Lebanese hospitals. Third, sole outcome investigated was the 30-day all-cause mortality, which may not be directly attributable to the *A. baumannii* infection. Finally, critical data, including the admitting diagnosis, laboratory findings, the severity of illness, follow-up culture results, and clinical response to treatment based on symptoms and signs monitoring, were not retrieved from the patients’ medical records. The absence of this information could potentially impact the depth of our analysis and limit our ability to establish causality. Therefore, a prospective multicenter longitudinal study with standardized measurements is needed to generate robust evidence.

## Conclusions

Carbapenem-resistant *A. baumannii* infections are highly prevalent in the study setting. Although all isolates were susceptible to colistin, the mortality rate of the infection was very high, which might be due to inappropriate initial antimicrobial treatment with the existing antimicrobial resistance, thrombocytopenia, and invasive mechanical ventilation. The study findings highlight the pressing need for collaborative efforts to address antimicrobial resistance in Lebanon. They emphasize the urgent need to implement comprehensive measures such as strict infection control protocols, annual nationwide surveillance programs, and effective antimicrobial stewardship programs at the national level, aiming to limit the unnecessary use of broad-spectrum antibiotics.

## Data Availability

The dataset presented in this article is available only upon reasonable request since it contains confidential information. Requests to access the datasets should be directed to the first author (r.itani@bau.edu.lb).

## Abbreviations

*A. baumannii*: *Acinetobacter baumannii*
AMR: Antimicrobial resistance
AOR: Adjusted odds ratio
ASP: Antimicrobial stewardship program
ESKAPE: *Enterococcus faecium, Staphylococcus aureus, Klebsiella pneumoniae, Acinetobacter baumannii, Pseudomonas aeruginosa,* and *Enterobacter* species
CI: Confidence interval
CRAB: Carbapenem-resistant *Acinetobacter baumannii*
CSAB: Carbapenem-susceptible *Acinetobacter baumannii*
FDA: Food and drug administration
IOC: Item-objective congruence
IRB: Institutional review board
MDR: Multidrug resistance
MDRAB: Multi-drug resistant *Acinetobacter baumannii*
PDR: Pandrug resistance
PDRAB: Pandrug resistant *Acinetobacter baumannii*
SD: Standard deviation
SE: Standard error
SPSS^®^: The Statistical Package for the Social Science
UOR: Unadjusted odds ratio
VAP: Ventilator-associated pneumonia
WHO: World Health Organization
XDR: Extensively drug resistance
XDRAB: Extensively-drug resistant *Acinetobacter baumannii*
